# Project for the National Program of Early Diagnosis of Endometrial Cancer Part I

**Published:** 2015

**Authors:** RE Bohîlțea, V Ancăr, MM Cirstoiu, V Rădoi, LC Bohîlțea, F Furtunescu

**Affiliations:** * “Carol Davila” University of Medicine and Pharmacy, Bucharest, Romania

**Keywords:** endometrial cancer, early diagnostic, endometrial hyperplasia, endometrial cancer screening, Lynch syndrome

## Abstract

**Rationale:** Endometrial cancer recorded a peak incidence in ages 60-64 years in Romania, reaching in 2013 the average value of 8.06/ 100,000 women, and 15.97/ 100,000 women within the highest risk age range, having in recent years an increasing trend, being higher in urban than in rural population. Annually, approximately 800 new cases are registered in our country. The estimated lifetime risk of a woman to develop endometrial cancer is of about 1,03%. Based on an abnormal uterine bleeding, 35% of the endometrial cancers are diagnosed in an advanced stage of the disease, with significantly diminished lifetime expectancy.

**Objective:** Drafting a national program for the early diagnosis of endometrial cancer.

**Methods and Results:** We proposed a standardization of the diagnostic steps and focused on 4 key elements for the early diagnosis of endometrial cancer: investigation of abnormal uterine bleeding occurring in pre/ post-menopausal women, investigating features/ anomalies of cervical cytology examination, diagnosis, treatment and proper monitoring of precursor endometrial lesions or cancer associated endometrial lesions and screening high risk populations (Lynch syndrome, Cowden syndrome).

**Discussion:** Improving medical practice based on diagnostic algorithms addresses the four risk groups, by improving information system reporting and record keeping. Improving addressability cases by increasing the health education of the population will increase the rate of diagnosis of endometrial cancer in the early stages of the disease.

**Abbreviations:** ACOG = American Society of Obstetricians and Gynecologists, ASCCP = American Society for Colposcopy and Cervical Pathology, PATT = Partial Activated Thromboplastin Time, BRCA = Breast Cancer Gene, CT = Computerized Tomography, IFGO = International Federation of Gynecology and Obstetrics, HLG = Hemoleucogram, HNPCC = Hereditary Nonpolyposis Colorectal Cancer (Lynch syndrome), IHC = Immunohistochemistry, BMI = Body Mass Index, INR = International Normalized Ratio, MSI = Microsatellites instability, MSI-H/ MSI-L = high (positive test)/ low (negative test) microsatellites instability, WHO = World Health Organization, PCR = Polymerase chain reaction, MRI = Magnetic Resonance Imaging, SGO = Society of Gynecologic Oncologists, SHG = Sonohysterography, SRU = Society of Radiologists in Ultrasound, TQ = Time Quick, BT = Bleeding Time, TVUS = Transvaginal ultrasound, USPIO = Ultrasmall superparamagnetic iron oxide

## Introduction

Based on the study of the current epidemiology and the critical analysis of the national and international practice guides, a list of problems has been identified, which were organized in a problems tree, for which an objectives tree analysis was conceived. The project proposed contains standardized diagnosis stages and diagnosis algorithms addressed to the four categories of population identified and considered to present a high risk of developing endometrial cancer, with the purpose of an early diagnosis of this pathology. Realizing the Project for the National Program of Early Diagnosis of Endometrial Cancer relies on the guidelines, recommendations and available protocols until present, formulated by the forums and specialty national and international societies, such as: IFGO (International Federation of Gynecology and Obstetrics), ACOG (American College of Obstetricians and Gynecologists), Society of Gynecologic Oncologists, World Health Organization, American Cancer Society, International Society of Ultrasound in Obstetrics and Gynecology, Romanian Society of Obstetrics and Gynecology. The theoretical background is based on specialty literature, guidelines and protocols which are currently applied, meta analyses, as well as randomized and controlled studies, which are well conceived, among which the following should be mentioned: the Clinical guide “Endometrial cancer”, elaborated by the Ministry of Public Health in Romania, the Obstetrics and Gynecology Counseling Commission of the Ministry of Public Health, the Obstetrics and Gynecology Commission of the College of Physicians in Romania and the Society of Obstetrics and Gynecology in Romania, “Endometrial cancer”, National Clinical Protocol (NCP-139) approved by the Experts Council of the Ministry of Health of the Republic of Moldova, in 2014, UpToDate, American Cancer Society Guidelines for Early Endometrial Cancer Detection: Update 2001; Cancer Screening in the United States 2013, Terms, definitions and measurements to describe the sonographic features of the endometrium and intrauterine lesions: a consensus opinion from the International Endometrial Tumor Analysis (IETA) group, 2010 [**2**]. 

The National Health Program represents a set of health services for a population with particular health (risk) problems and having as a purpose the accomplishment of some objectives set in relation to those problems [**3**]. The Program puts together in a coherent and organized manner the three main elements of a health problem: objectives, activities, and resources [**4**]. There is a specific legislative framework for the implementation of national health programs in Romania, according to which these represent the frame for the implementation of the politics objectives and the public health strategy by the Ministry of Health, as a central authority in the field (Law no. 95/ 2006 regarding the Reform in Public Health, published in the Official Monitory, Part I, no. 372 from 28/04/2006 with the further modifications and additions). Therefore, the national health programs answer to some public health problems, which are considered to have priority, due to the frequency of the affections or the gravity of the consequences as far as the person or the health system is concerned. The Programs are addressed to the risk population and are oriented towards the promotion of health, the prevention of disease, and the prolonging of a quality life. The type of Program proposed by the present research, can be implemented as a national health program or by remodeling the medical services provided by the stable entities in the system of social health insurances system (family physicians, specialty ambulatories, hospitals), or combined. The following steps have been made to conceive the National Program: identifying the health problems connected to the analyzed pathology, defining the purpose of the Program and the general and specific objectives, identifying the feasible strategies for the goals achievement, identifying the activities of the Program and the necessary tools, defining the monitorization and evaluation indicators specific to the Program.

## Results and Discussions

**Fig. 1:**
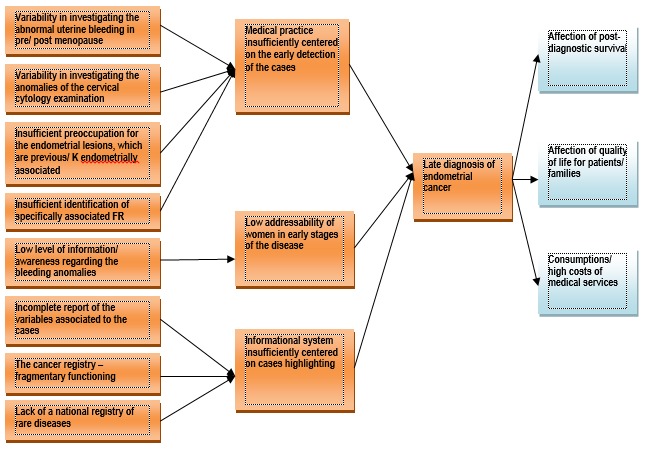
The problems tree regarding the endometrial cancer in

**Fig. 2:**
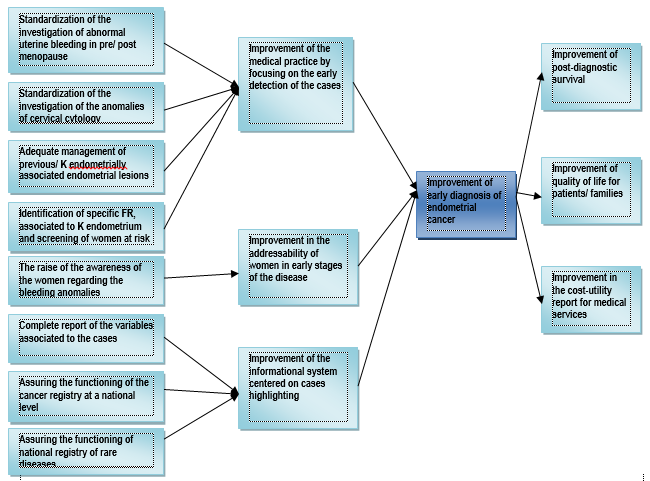
The objectives tree regarding endometrial cancer in Romania

Taking into account the late diagnosis of endometrial cancer in at least a third of the cases, the identification of its determinants has been tried (the lack of real national statistical indicators, the report of the cases having a common diagnosis without etiological individualities, the absence of a computer system and of national databases, the lack of the updating of the clinical guide elaborated by the Ministry of Health, the centralized malfunction of the National Cancer Registry and the National Registry of Genetic Diseases, the variability of the reports of endometrial ultrasonographic examination as a selection method for the patients with high risk, the lack of use of reliable endometrial biopsy methods, the insufficient involvement of the family physician in the detection of risk factors and the abnormal uterine bleeding as well as in the informing of the patients and the low level of the national sanitary education) (**Fig. 1**) and offering with the help of the Program solutions with the purpose of improving the process of an early diagnosis of endometrial cancer (the standardization of the pre/ post menopause abnormal uterine bleeding at the level of hospital, specialty ambulatory and family physician; the standardization of cervical cytologic examination anomalies investigation; adequate management of endometrial lesions precursor/ associated to endometrial cancer; identification of high risk factors associated to endometrial cancer and screening of women in the high risk group; improvement of the addressability to women in early stages of the disease and the improvement of the information system in order to highlight the path of the cases) (**Fig. 2**). 

**Definitions and terms**

***Abnormal uterine bleeding*** in premenopausal women represents any intermenstrual or menstrual bleeding at an interval shorter than 21 days, with the loss of a blood volume of more than 80 ml, or which lasts for more than 7 days. Any uterine bleeding of a postmenopausal woman, except for the menstrual bleeding determined by the sequential hormonal therapy administered post menopause, is considered abnormal. 

***Endometrial polyps*** are hyperplasic proliferations of the endometrial glands and of the stromal tissue, having an own vascular pedicle. 

***Endometrial hyperplasia*** represents an exaggerated proliferation of the endometrium, which is characterized by the growth of the glands/ stroma ratio, the variability of the forms and dimensions of the proliferated glands and the possibility of cytologic atypia and progression to endometrial cancer. 

***Endometrial cancer*** represents the malign tumor of the endometrium characterized by an autonomous growth, local invasion with the destruction of the normal tissues and spreading through multiple metastases, producing a progressive disease, which ends with death. 

**Risk factors**

Endometrial carcinomas present two different categories with regard to incidence, estrogen-dependency, and clinical behavior. Type I includes the grade 1 and 2 endometrial tumors, which represent 80-90% of the endometrial carcinomas, a characteristic of this type being the favorable prognosis, the dependency on estrogens and the etiopathogenic relation with intraepithelial neoplasia or the endometrial hyperplasia with or without atypia. Type 2 is represented by 10-20% of the endometrial cancers and includes grade 3 endomerioid and non-endometrioid tumors, respectively serous, with clear, mucinous, squamous cells and transition, mesonephric and undifferentiated cells, being most often high-grade tumors with an unfavorable prognosis, an inconstant association of the estrogenic stimulus and with a rarely identified precursor lesion [**5**-**7**]. 

The most important risk factor for type I is the long-term exposure to the endogenous or exogenous estrogen stimulus, which is improperly antagonized from a progesterone point of view. The exogenous estrogen excess results from the estrogen hormone substitution therapy and the tamoxifen therapy, while the excessive endogenous exposure results from obesity, anovulatory menstrual cycles, or estrogen secreting tumors.

1. ***Age*** represents an independent risk factor; the risk of a woman aged 50-70 years of developing endometrial cancer is of 1,4% [**8**]. 

2. ***The systemic estrogen therapy*** raises the incidence of endometrial cancer with a relative risk that varies between 2 and 10, depending on the dose and the period of administration [**8**-**10**]. 

3. ***Tamoxifen therapy*** raises the risk of endometrial cancer with a rate equal to 2, depending on the period of administration [**8**]. Tamoxifen raises the risk of endometrial carcinoma appearance and of uterine sarcoma in post menopause period, without the existence of conclusive data for the pre-menopause women [**11**,**12**].

4. ***Late menopause*** appears after 55 years and associates a relative risk equal to 2 [**8**,**13**]. 

5. ***Premature menarche*** raises the risk of endometrial cancer; however, the relative risk is not available [**8**,**13**].

6. ***Nulliparity*** induces a raise of the basic risk with 2 times [**8**,**13**]. 

7. ***Syndrome of polycystic ovaries*** diagnosed according to Rotterdam criteria, determines chronic anovulation, raising the risk of endometrial cancer with a coefficient that equals with 3 [**8**,**14**-**16**]. 

8. ***Obesity*** represents an important risk factor for the endometrial cancer. Women with a BMI ≥ 30 kg/ m2 present a basic risk of 2-4 times higher, endometrial cancer appearing in young ages and having a higher mortality rate [**8**,**17**,**18**]. 

9. As an individual factor, ***diabetes*** determines a relative risk of endometrial cancer, which equals with 2 [**8**,**19**-**25**]. 

10. ***Hypertension*** raises the risk of endometrial cancer as an individual factor and as a comorbidity element of obesity and diabetes; however, the relative risk is not available [**8**,**19**,**26**,**27**]. 

11. ***Estrogen-secreting tumors*** determine the development of endometrial cancer; however, the relative risk is not available [**8**,**28**]. 

12. ***Lynch syndrome (HNPCC)*** is a genetic affection characterized by a dominant autosomal transmission, which has the mutation of one of the genes responsible for the DNA repair errors crossing as a base (MMR): MSH2, MLH1, MSH6, PMS2, and which determines a risk of appearance of endometrial cancer between 27 and 71% during lifetime. In this syndrome, cancer appears at an early age and has multiple localizations, usually the colon and the endometrium [**29**,**30**]. 

13. ***Cowden syndrome*** is a genetic affection with a dominant autosomal transmission which has as a basis a mutation of PTEN suppressor gene and which determines a risk of appearance of endometrial cancer of 13-19% during lifetime [**8**,**31**,**32**].

14. ***Family history of endometrial, ovarian, breast and colon cancer*** represent a potential risk factor for the appearance of endometrial cancer; however, the relative risk is not available [**8**,**33**]. 

Type II endometrial carcinoma has a medium diagnosis age of 65 years, it appears in women with a history of one or more pregnancies, black race and obesity raising the predisposition of developing this type of cancer. The personal history of breast cancer is correlated with a high risk of developing serous uterine carcinoma [**34**-**36**]. 

**The activities of the Program**

**1. Defining the population with a high risk [**37**], who has to be investigated in order to early diagnose endometrial cancer**: 

- Post menopause women with an uterine bleeding regardless the volume, duration or frequency (sanguinolent leucorrhea/ spotting/ metrorrhagia); 

- Post menopause women with an endometrial thickness measured by a transvaginal ultrasound of > 4mm, regardless the presence/ absence of bleeding;

- Women aged between 45 years and menopause who present any abnormal uterine bleeding, including an intermenstrual bleeding which appears during the ovulation period;

- Women aged between 45 years and menopause who present an abnormal uterine bleeding from the point of view of the frequency (a time interval of < 21 days between the starting of the bleeding of two consecutive menstrual cycles), quantity (total volume of blood loss of > 80 ml), or duration (> 7 days);

- Women aged under 45 years who present any abnormal persistent bleeding, which appeared as a result of the improper progesterone non-antagonized estrogenic exposure (obesity, anovulatory menstrual cycles); 

- Women aged under 45 years who present an abnormal uterine bleeding and failure of medication therapy;

- Women aged under 45 years who present an abnormal uterine bleeding while under tamoxifen treatment, or without bleeding but with a high risk of developing endometrial cancer (Lynch syndrome, Cowden syndrome); 

- Premenopausal women who present anovulatory amenorrhea of > 6 months and an endometrial thickness measured by a transvaginal ultrasound of > 7mm;

- Women with cervical cytology presenting atypical endometrial glandular cells (AGC); 

- Women with cervical cytology presenting atypical glandular cells (AGC) of all the subcategories beside the endometrial ones, aged over 35 years; 

- Women with cervical cytology presenting AGC of all the subcategories beside the endometrial ones, with a high risk of developing endometrial cancer (abnormal uterine bleeding/ risk factors);

- Women with cervical cytology presenting benign aspect endometrial cells, aged ≥ 40 years and having an abnormal uterine bleeding/ risk factors of developing endometrial cancer; 

- Women with an endometrial pathology (ex. endometrial hyperplasia) need monitoring; 

- Women with Lynch syndrome or Cowden syndrome present a high risk of developing endometrial cancer and need screening. 

**2. Endometrial cancer screening**

**Standard**

The routine screening for endometrial cancer is not recommended for the general population. Women with high risk or moderately asymptomatic, who are not exposed to routine screening because there are not enough high quality data to support the efficiency of screening in this population group, with the purpose of reducing endometrial cancer mortality rates. This recommendation includes women with risk factors for endometrial cancer, except for the ones with Lynch syndrome and Cowden syndrome, who present a higher risk, of 27-71%, and respectively of 13-28% of developing endometrial cancer during lifetime, compared to 1,4-2,6% - the risk of the general population, and who, due to this reason, benefit from screening. 

**Standard**

The screening for endometrial cancer of asymptomatic women with a genetic diagnosis of Lynch syndrome (HNPCC)/ family history of Lynch syndrome (HNPCC)/ family aggregation of colorectal or endometrial cancer without a genetic diagnosis of Lynch syndrome (HNPCC), consists in an endometrial biopsy performed annually starting with the age of 30-35 years or 5-10 years before the earliest age of diagnosing a cancer form associated to Lynch (HNPCC) Syndrome in the family [**38**,**39**].

**Recommendation**

The performance of the transvaginal ultrasound examination is complementary to the endometrial biopsy and is performed annually, starting with the same age. For the asymptomatic women with a family history of Lynch syndrome or a family aggregation of colorectal or endometrial cancer without a genetic diagnosis of Lynch syndrome (HNPCC), genetic examination and genetic testing of MMR gene mutations are recommended [**40**].

**Option**

Endometrial biopsy can be a suction biopsy, cervical dilation, and uterine curettage or centered by hysterectomy followed by uterine curettage.

For the patients with Cowden syndrome, there are no specific certificated regulations, but the high risk of endometrial cancer development which they present, justifies the application of screening through endometrial biopsy starting with the age of 35-40 or 5 years earlier to the age of diagnosing the first case of endometrial cancer in the family and annual transvaginal ultrasound in the post menopause period [**39**]. 

As far as tamoxifen is concerned, an important risk factor for endometrial cancer, until present, there are no recommendations of the experts for routine screening of asymptomatic patients. However, any abnormal uterine bleeding in a patient under tamoxifen therapy imposes an investigation of endometrial cancer exclusion. 

**Standard**

The premenopausal asymptomatic patients under tamoxifen therapy do not need a special monitoring supplementary to the routine gynecological evaluation. They must by clinically examined annually. The patients with abnormal uterine bleeding under tamoxifen therapy must be examined by performing an endometrial biopsy [**41**,**42**]. 

**Recommendation**

The duration of tamoxifen therapy must be limited to 5 years. The patients must be informed regarding the high risk of developing endometrial cancer and must be counseled with regard to the unexpected appearance of symptomatology near menopause, to be encouraged to ask for medical assistance in case of any abnormal uterine bleeding [**41**,**42**]. 

**3. The diagnosis of endometrial cancer**

The early diagnosis of endometrial cancer is based on 4 elements:

1. Investigation of the abnormal bleeding during pre/ post menopause; 

2. Investigation of the particularities/ anomalies of the cervical cytology examination; 

3. Diagnosis, treatment and adequate monitorization of the endometrial lesions precursor/ associated to endometrial cancer;

4. Screening of the high-risk population (Lynch syndrome, Cowden syndrome). 

The early diagnosis of endometrial cancer can be accomplished in the following stages: 

I. Stage of initial evaluation

It is addressed to the high-risk population of developing endometrial cancer, which was previously defined. The purpose is the selection of cases likely to develop endometrial cancer, that need supplementary investigations for confirmation. 

**Standard**

The examination is made up of medical history, general and gynecological clinical examination and initial laboratory investigations. 

The medical history must contain:

• Medical physiological personal history such as age, number of births and their accomplishments, the number of abortions, the menstrual cycle history from the point of view of frequency, regularity, volume, duration and phenomena occurring during the menstrual cycle, date of the last menses, the age menarche and menopause installed, history of cervical cytology; 

• Medical pathological personal history (hypertension, obesity, diabetes, coagulation thyroid urinary or intestinal disorders), surgeries, gynecological examinations (the characteristics of abnormal uterine bleeding, results of the endometrial biopsy, if it was previously undergone, leucorrhea, pelvic/ abdominal pain, pelvic inflammatory affections, uterine or annex tumors), treatments done with effects on the hemostatic or endocrine function (anticoagulants, progesterone therapy, hormone substitution therapy during menopause, combined oral contraception, tamoxifen, thyroid hormones);

• family medical history: family history of cancer with the age of cancer diagnosis and the family degree of relationship, for breast, endometrial, ovarian, colorectal, gastric, small intestine, biliary, pancreatic, upper urological tract, cerebral (gliomas), sebaceous glands cancer; maternal gynecologic history, congenital anomalies, genetic diseases, hemorrhagic diatheses, diabetes. 

• identification of risk factors for endometrial cancer.

General physical examination on devices and systems should contain general state, height, weight, BMI, examination of teguments and mucosa, of musculo-adipose, ganglio-lymphatic, osteoarticular, respiratory, cardiovascular, digestive, urinary, nervous and endocrine systems and also the breasts examination systematically done by inspection and palpation.

The physical gynecological examination should contain the following elements: valves examination, which evidences the sources of bleeding (indirectly uterine bleeding) at the level of the perineum, vulva, vagina and cervix, modifications which have an inflammatory, lesion or tumoral character, the presence of foreign bodies, the vaginal examination which evidences the position, mobility, dimensions, consistency, sensitivity at the level of the cervix, uterus and annexes, as well as the infiltrative modifications of the vagina and vaginal annexes, and the rectal examination which evidences the relations between the internal genital organs with the pelvic structures nearby.

The laboratory tests should contain the following elements: β hCG urinary+/ -serum dosage, complete CBC, coagulation tests (APTT, INR, TS, TQ, fibrinogen), Babeş Papanicolau cytological examination +/ - high risk HPV testing, bacteriological examination of vaginal secretion.

II. Stage of etiological evaluation 

It is addressed to the population susceptible of endometrial cancer. 

The purpose is the selection of the cases suspect of endometrial cancer, which need histopathological confirmation.

**Standard**

It contains imagistic explorations and endometrial biopsy.

Transvaginal ultrasound (TVUS) represents the first line of imagistic exploration of the abnormal uterine bleeding, being a non-invasive, cost-efficient method, which is reliable, easily tolerated by the patient, very often used due to the introduction of ultrasound in Obstetrics and Gynecology in the curriculum of the field and by the extended equipping with performing devices of most of the medical units. Postmenopausal TVUS differentiates the patients with high risk of endometrial cancer (endometrial thickness of > 4mm) of the ones who do not need supplementary investigations (endometrial thickness of ≤ 4mm) [**43**-**52**]. The etiological structural anomalies of the abnormal uterine bleeding are differentiated in pre menopause [**53**]. The random ultrasound discovery of the endometrial thickness of > 4mm in postmenopausal asymptomatic women imposes an endometrial biopsy. The high risk of ovarian cancer in patients with Lynch syndrome justifies the association of transvaginal ultrasound with screening by biopsy indicated for endometrial cancer. While monitoring endometrial hyperplasia in pre menopause, sensitivity and specificity are reduced, but in post menopause, they represent the first line of exploration both in the case of hyperplasia monitorization during the treatment and in the case of diagnosing the relapse of endometrial polyps. In the anomalies of the cytologic examination, TVUS represents the second line of exploration after endometrial biopsy.

Suction biopsy represents the first line of biopsy investigation of abnormal uterine bleeding. The method is minimally-invasive, cost-efficient, can be performed in the ambulatory, it is well accepted by the patients, does not require anesthesia and has a high accuracy for endometrial cancer, the diagnosis sensitivity of the suction biopsy is of approximately 67-98% and the specificity of 100% for cases in which enough tissue is obtained and the pathological endometrial process is global. The positive result is the diagnosis for endometrial cancer, but the negative result does not exclude the disease. The false negative results reported in a variable percent of 7-33% are due to the focal/ limited lesions to < 50% of the endometrial surface. The biopsy material is insufficient in 13-22% of the cases due to the focal lesions and the postmenopausal status [**54**-**62**]. The negative suction biopsy through an insufficient material imposes the repeating of the suction biopsy or dilation and curettage. The failure of two consecutive suction biopsies imposes the endometrial biopsy by dilation and curettage or the association hysteroscopy-targeted biopsy with post hysteroscopy uterine curettage, which represents the optimal method of diagnosis of focal lesions [**63**,**64**]. The persistent/ recurrent bleeding after an endometrial suction biopsy with a benign result needs the repeating of the procedure at 3-6 months.

With a diagnostic sensitivity for hyperplasia and cancer of 92-94% in cases of diffuse lesions, although only 30-60% of the endometrial surface is diffused, and being limited in exo/ endophytic focal lesions, biopsy by dilation and curettage, the gold standard of endometrial cancer diagnosis, has an increased accuracy (77%) in identifying the tumor degree in comparison with the suction biopsy (58%) [**64**,**65**] and is indicated in the following cases: 

• management of abnormal acute uterine bleeding or the temporary management of prolonged excessive bleeding unresponsive to hormonal therapy;

• non-diagnostic suction biopsy in patients with a high risk of endometrial cancer; 

• suction biopsy with insufficient material for the histopathological study; 

• suction biopsy with a negative result but with persistent bleeding; 

• endometrial hyperplasia for the exclusion of endometrial cancer; 

• cervical stenosis which needs preparation procedures and cervical dilation;

• focal endometrial pathology; 

• endometrial cancer with the desire to preserve fertility, anterior to the start of medicine therapy and during its monitoring; 

• the preoperatory exclusion of endometrial cancer in leiomyomas treatment; 

• the need for a concomitant procedure, such as hysteroscopy or laparoscopy; 

• intolerance of suction biopsy (pain, anxiety). 

Hysteroscopy represents an invasive procedure which while used does not overcome the diagnosis accuracy of dilation and curettage (3,4% false negative, specificity of 86% in post menopause), only while associated with it. It also needs a qualified staff and technical equipment, inducing the raise of the exploratory costs. It is indicated as a method of choice in the biopsy exploration of focal lesions that were found at the ultrasound examination. It is recommended that the exploration by curettage of the entire endometrial surface (SGO) is completed.

**Recommendation:** The use of Doppler function raises the specificity of echographic examination and it is recommended to be used as a routine analysis in gynecological ultrasound examination. The instillation of saline substance is also recommended as a routine action during the endometrium ultrasound investigation, directing the optimum modality of endometrial biopsy: suction or dilation biopsy and uterine curettage in global endometrial lesions, hysteroscopy with targeted biopsy and post hysteroscopic uterine curettage in focal lesions. SHG or hysteroscopy are indicated especially in case in which by transvaginal conventional ultrasound, the endometrium cannot be evaluated or the existence of focal endometrial pathology is suggested. The virgo intact patients or the ones with inconclusive results of the imagistic and biopsy investigations according to the stage, may benefit from an MRI investigation. The ovarian ultrasound evaluation and the suprarenal glands investigation is recommended in post menopause women who do not present obvious signs hyperestrogenism. The positive history by coagulopathies screening needs tests for hereditary coagulopathies. The anovulatory disorders need hormonal tests.

**Option:** In case ultrasonography or suction biopsy tools lack, the first choice of investigation is dilation and uterine curettage.

III. Stage of histopathological confirmation

It is addressed to cases that are suspected of endometrial cancer, which need a histopathological confirmation.

The purpose is obtaining the clear diagnosis of endometrial cancer, establishing the histopathological class and degree, establishing the presence of microsatellites instability (MSI) and the structural loss/ alteration of proteins encoded by MMR genes. 

The diagnosis of endometrial cancer is a histopathological one, which can be reached based on the analysis of the fragments resulted from the suction biopsy, uterine curettage, hysteroscopy or based on the investigation of the hysterectomy piece.

**Standard:** It contains histopathological diagnosis techniques of the fragments extracted by performing a biopsy and processed in paraffin/ frozen (by cryogenesis). 

**Recommendation:** The testing of MSI by PCR and the immunohistochemical testing of proteins coded by MMR genes, followed by monogenic sequencing, identifies the endometrial cancers in Lynch syndrome. Testing is recommended in all the patients with endometrial cancer, being necessary in patients with endometrial cancer which was diagnosed before 50 years old, Lynch-synchronic/ methachronic associated tumors, endometrial cancers with tumoral or peritumoral lymphoid infiltrate, histologically undifferentiated tumors or with origin in the inferior uterine segment which appeared before 60 years old, as well as in patients with a family history which suggests this genetic syndrome (SGO). 

**Optional:** Electronic microscopy. 

IV. Stage of evaluation of the local spreading/ metastasis of the disease and the biological and functional pre therapeutic status.

Is addressed to patients diagnosed with endometrial cancer. 

The aim is to evaluate the general health state of the patient and the biological status in a preoperative balance. What is also important is the evaluation anterior to the operatory staging of the disease spreading and the presence/ absence of distant metastases in order to decide upon the surgical therapeutic plan and the supply of a reference level of CA-125 tumor marker for the post-therapeutic follow-up of the disease. 

**Standard:** The physician should highlight that the evaluation of the biological and functional pre therapeutic status should contain the following minimal compulsory investigations: 

Preoperatory laboratory tests: 

• Complete HLG 

• Coagulation tests: PATT, INR,TS,TQ, fibrinogen

• Biochemical tests: glucose level, TGO, TGP, bilirubin, urea, creatinine

Preoperatory functional balance: 

• Electrocardiogram

• Lung radioscopy 

• Lung functional explorations 

• Abdominal pelvic ultrasound 

While planning the standard surgical staging, the imagistic preoperatory determination of the myometrial and cervical invasion is recommended but not compulsory. In exceptional cases in which the staging must be clinically established, respectively in patients with stage I grade 1 endometrial cancer who want to preserve fertility, and also in patients who cannot be operated, the magnetic resonance imaging (MRI) with contrast substance represents the best radiological method of establishing the myometrial and cervical spreading of endometrial (80-90% sensitivity for myometrium and respectively 56-100% for cervix), compared with MRI without contrast, ultrasonography or CT: the evaluation of the ganglionar metastases are optimally realized by MRI (USPIO), superior to the CT evaluation or positron emission tomography (PET), with or without CT. The accuracy of CT diagnosis in the evaluation of the distant metastases of stage III and IV is of 86%. The depth of the invasion is not predictable through endometrial biopsy.

**Recommendation:** The evaluation of the local spreading of the disease and the presence or absence of distant metastases significantly contributes to the operative planning and the therapeutic conduct. MRI routine pre-operatory evaluation with contrast substance is recommended. 

According to the symptoms, the next supplementary investigations might be necessary: cystoscopy, urography, colonoscopy, bone scintigraphy.

CA-125 > 40UI/ ml has a sensitivity of 78% and a specificity of 81% in signaling the presence of ganglionar metastases [**50**,**66**-**70**]. The initially raised levels are used in the post-therapeutic monitorization of the patients.

The family history suggestive for Lynch syndrome recommends the genetic examination and the undergoing of genetic testing for MMR genes mutations, because the risk of synchronous tumors found intraoperatively in patients with this syndrome is high. The psychological consultation is also recommended preoperatively. 

**Option:** Transvaginal ultrasound with Doppler and 3D evaluation proved to have a sensitivity and specificity compared to the ones of MRI in evaluating the cervical and myometrial invasion when the MRI is not available. SHG has a high diagnostic accuracy in appreciating the local spreading of endometrial cancer, compared to TVUS. 

V. Staging endometrial cancer

**Standard:** According to IFGO/ AJCC Agreement, the correct staging of endometrial carcinoma is done by surgery, while using TNM classification and the degree of histopathological differentiation. The standard procedure of staging the endometrial carcinoma is represented by total extrafascial hysterectomy with bilateral adnexectomy and dissection of the pelvic and para-aortic ganglia, done by laparotomy, robot-assisted laparoscopic surgery, or conventional laparoscopy. The complete staging includes peritoneal biopsies from the areas suspect of metastasis and the peritoneal suction or lavage, even if it does not represent a part of the new IFGO staging anymore. Cytoreduction of clear metastases is performed. In the absence of obvious secondary determinations, omentectomy is indicated in cases of serous carcinomas and clear cell carcinomas. Taking into account the fact that the presence of the extrauterine disease and especially the metastasis of pelvic and para-aortic ganglia represent the most important prognosis factor for endometrial cancer, as well as the fact that ganglia dissemination depends on the stage and degree of the tumor and the para-aortic lymphatic ganglia can be positive in the absence of positive pelvic lymphatic ganglia, IFGO recommends the mandatory intraoperative evaluation of both the pelvic and the para-aortic ganglia, without which staging is considered incomplete; the evaluation method (biopsy and excision) and the spreading are not mentioned. Palpation and ganglionar examination are not methods of appreciating the metastasis. Lymphadenectomy is the most comprehensive and precise evaluation method of the patients with an apparent stage I endometrial cancer. Spread pelvic and para-aortic lymphadenectomy is preferable to ganglionar biopsy. 

**Recommendation:** In the presence of any of the following high risk factors for ganglia metastasis, the resection of pelvic and para-aortic ganglia is indicated: serous carcinoma, clear cell carcinoma, high histological degree, myometrial invasion > 50%, tumor > 2m or which fills up the endometrial cavity [**54**]. Studies affirm that the resection of para-aortic ganglia is indicated in patients with high and intermediate risk of disease recurrence, the patients with low risk not being demonstrated to benefit from a prognosis from performing the spread lymphadenectomy. 

**Standard:** The hysterectomy piece must be sectioned in the operating room for staging and adopting the correct therapeutic conduct. Establishing the depth of the myometrial invasion through macroscopic evaluation has a sensitivity of 75% and a specificity of 92% [**71**]. The conformity of the examination on frozen sections of the maximum area of myometrial invasion, having a final histopathological result, varies between 85 and 98,7% [**72**,**73**].

The postoperatory histopathological result should contain: 

• type of tumor cells; 

• histopathological degree of tumor;

• depth of the myometrial tumor invasion; 

• cervical tumor invasion; 

• lymphovascular space invasion of tumor; 

• biopsied/ excised lymph invasion of tumor; 

• positivity/ negativity of biopsied fragments;

• cytology of ascitic fluid or peritoneal lavage (even if it is not included in the staging process in the new classification);

• MSI-H/ L and/ or the result of the immunohistochemical study of proteins coded by MMR genes.

**Recommendation:** Patients with stage IA first degree, who wish to preserve fertility, should benefit from staging and a standard surgical treatment, after the familial planning discussion, even in the case of tumor regression demonstration.

**4. Investigating pre/ post menopause abnormal uterine bleeding**

Premenopause abnormal uterine bleeding is defined by IFGO 2011 [**74**] and contains the following entities: 

• chronic abnormal uterine bleeding represents the bleeding which comes from the endometrium level, it is abnormal as far as the volume and regularity and/ or duration is concerned, and characterizes most of the menses in the last 6 months; 

• the abnormal acute uterine bleeding represents an abundant bleeding episode, evaluated by the physician as being severe enough to require an immediate intervention in order to prevent the continuous loss of blood; it can happen as a result of a chronic abnormal uterine bleeding or presenting a history of normal menses; 

• intermenstrual bleeding represents the bleeding between two clearly defined menses from the cyclic and predictability point of view, randomly or rhythmically repeated in the same period of the menstrual cycle; IFGO recommends the replacement of the term metrorrhagia with intermenstrual bleeding. 

The post menopause abnormal uterine bleeding represents any uterine bleeding, regardless the volume, of a postmenopausal woman, except for the cyclical bleeding determined by the sequential hormonal therapy administered postmenopausal. 

**Standard:** Pregnancy is excluded, premenopausal or menopausal status is established, together with the source of uterine bleeding by excluding the extrauterine genital bleedings (tubal, cervical, vaginal, vulvar), of urinary or gastrointestinal tract, by investigating the patient history, performing a general physical examination and initial laboratory tests during the initial stage.

The cases that are suspect of endometrial cancer, respectively the premenopausal and post menopause abnormal uterine bleedings go in the stage of etiologic evaluation. The abnormal acute uterine bleedings or the hemodynamic instability implies the patient’s transfer to an emergency hospital in order to perform a hemostatic and biopsy uterine curettage. 

According to PALM-COEIN classification, the etiological causes of abnormal uterine bleeding can be structured as it follows: polyp (P), adenomyosis (A), leiomyoma (L), malignity or endometrial hyperplasia (M) and independent of structural anomalies, respectively coagulopathies (C), ovulatory dysfunctions (O), endometrial dysfunctions (E) and iatrogenic causes (I); a separate class contains unclassified entities (U). The category of leiomyomas is divided in submucous leiomyomas and leiomyomas which do not leave a print on the endometrial cavity (IFGO Menstrual Disorders Group, 2011). 

**Fig. 3:**
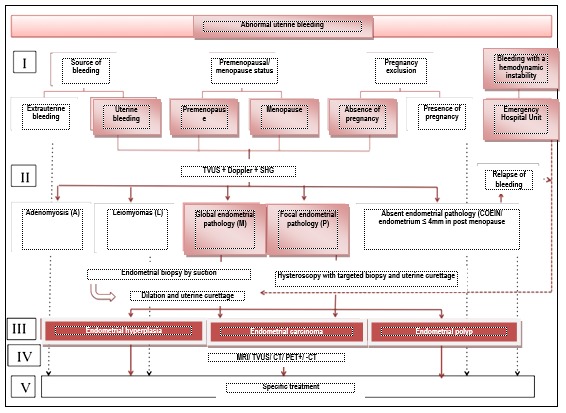
Diagnosis algorithm of abnormal uterine bleeding

**Standard:** The etiologic diagnosis of the structural causes has as a base transvaginal Doppler ecography and saline solution instillation during sonohysterography and endometrial biopsy by endometrial suction/ biopsy by dilation and curettage/ targeted hysteroscopic endometrial biopsy +/- endometrial biopsy by post hysteroscopy uterine curettage (**Fig. 3**). 

TVUS differentiates the etiological structural classes of bleedings and guides towards the optimal way of undergoing an endometrial biopsy: by hysteroscopic visualization and targeted biopsy, followed by uterine curettage of the entire endometrial cavity in the focal pathology or by suction or dilation and uterine curettage in global endometrial pathology. 

Abnormal uterine bleeding appears in 4-11% of postmenopausal women, among whom 10% due to endometrial cancer. Abnormal uterine bleeding with an endometrial thickness of ≤ 4 mm, evaluated on ultrasound, does not require supplementary investigations (endometrial cancer risk 1/ 917 cases, method sensitivity 96%, false negative rate 0,25-0,5%), TVUS application as a first line investigation method, reduces the number of applying useless biopsies with over 40% [**21**] (ACOG and SRU); and the recurrence/ persistence of bleeding impose an endometrial biopsy. In case of evidencing intracavity liquid by TVUS in menopause, the following should be excluded: endometrial cancer with a summed endometrial thickness of > 4mm or the evidencing of a focal lesion and cervical cancer as a cause of cervical stenosis that led to liquid accumulation. The prioritization of the patients with a high risk of endometrial cancer according to ultrasound criteria, for the urgent histopathological confirmation, includes the BMI together with the Doppler score, endometrial thickness, and interruption of B mode exploration junction and the surface irregularity in sonohysterography, in the prediction risk score for endometrial cancer (Dueholm 2014). By using these parameters, a score of ≥ 4 identifies the endometrial cancer with a sensitivity of 91% and a specificity of 94%. 

The abnormal uterine bleeding in post menopause needs an endometrial biopsy in the following cases: 

• endometrial thickness (TVUS) > 4mm;

• the endometrium cannot be clearly visualized by TVUS+/-SHG;

• endometrial thickness (TVUS) ≤ 4 mm but with persistent/ recurrent bleeding.

Endometrial biopsy is undergone by one of the 3 methods by respecting the indications of the etiological evaluation stage. The histopathological confirmation of the endometrial carcinoma is followed by the stage of evaluation of the local spreading/ disease metastasis and of the biological and functional pre-therapeutic status, then by IFGO and TNM staging of the endometrial cancer.

**To be continued in Journal of Medicine and Life, vol. VIII, issue 4, 2015**.

## References

[1] Bohîlțea RE, Furtunescu F, Dosius M (2015). Evaluation of endometrial cancer epidemiology in Romania. JML.

[2] Leone FPG, Timmerman D, Bourne T (2010). Terms, definitions and measurements to describe the sonographic features of the endometrium and intrauterine lesions: a consensus opinion from the international endometrial tumor analysis (IETA) group. Ultrasound Obstet Gynecol.

[3] Pineault R, Daveluy C (1986). La planification de la sante – concepts, methods, strategies. Agence D’Arc INC.

[4] Mincă DG, Marcu MG (2005). Sănătate publică şi management sanitar, note de curs pentru învăţământul postuniversitar.

[5] Sienko AE (2012). Advances in surgical pathology. Endometrial Cancer.

[6] Bokhman JV (1983). Two pathogenetic types of endometrial carcinoma. Gynecol Oncol.

[7] Felix AS, Weissfeld JL, Stone RA (2010). Factors associated with Type I and Type II endometrial cancer. Cancer Causes Control.

[8] Smith RA, von Eschenbach AC, Wender R American Cancer Society Guidelines for Early Endometrial Cancer Detection: Update 2001.

[9] Henderson BE (1989). The cancer question: an overview of recent epidemiologic and retrospective data. Am J Obstet Gynecol.

[10] Beral V, Bull D, Reeves G (2005). Million Women Study Collaborators. Endometrial cancer and hormone-replacement therapy in the Million Women Study. Lancet.

[11] Iqbal J, Ginsburg OM, Wijeratne TD (2012). Endometrial cancer and venous thromboembolism in women under age 50 who take tamoxifen for prevention of breast cancer: a systematic review. Cancer Treat Rev.

[12] Davies C, Godwin J, Gray R (2011). Early Breast Cancer Trialists’ Collaborative Group (EBCTCG). Relevance of breast cancer hormone receptors and other factors to the efficacy of adjuvant tamoxifen: patient-level meta-analysis of randomised trials. Lancet.

[13] McPherson CP, Sellers TA, Potter JD (1996). Reproductive factors and risk of endometrial cancer. The Iowa Women's Health Study. Am J Epidemiol.

[14] Hardiman P, Pillay OC, Atiomo W (2003). Polycystic ovary syndrome and endometrial carcinoma. Lancet.

[15] Gadducci A, Gargini A, Palla E, Fanucchi A, Genazzani AR (2005). Polycystic ovary syndrome and gynecological cancers: is there a link?. Gynecol Endocrinol.

[16] Giudice LC (2006). Endometrium in PCOS: Implantation and predisposition to endocrine CA. Best Pract Res Clin Endocrinol Metab.

[17] Pellerin GP, Finan MA (2005). Endometrial cancer in women 45 years of age or younger: a clinicopathological analysis. Am J Obstet Gynecol.

[18] Fader AN, Arriba LN, Frasure HE, von Gruenigen VE (2009). Endometrial cancer and obesity: epidemiology, biomarkers, prevention and survivorship. Gynecol Oncol.

[19] Furberg AS, Thune I (2003). Metabolic abnormalities (hypertension, hyperglycemia and overweight), lifestyle (high-energy intake and physical inactivity) and endometrial cancer risk in a Norwegian cohort. Int J Cancer.

[20] Noto H, Osame K, Sasazuki T, Noda M (2010). Substantially increased risk of cancer in patients with diabetes mellitus: a systematic review and meta-analysis of epidemiologic evidence in Japan. J Diabetes Complications.

[21] Zhang Y, Liu Z, Yu X, Zhang X, Lü S, Chen X, Lü B (2010). The association between metabolic abnormality and endometrial cancer: a large case-control study in China. Gynecol Oncol.

[22] Soliman PT, Wu D, Tortolero-Luna G, Schmeler KM, Slomovitz BM, Bray MS, Gershenson DM, Lu KH (2006). Association between adiponectin, insulin resistance, and endometrial cancer. Cancer.

[23] Zendehdel K, Nyrén O, Ostenson CG, Adami HO, Ekbom A, Ye W (2003). Cancer incidence in patients with type 1 diabetes mellitus: a population-based cohort study in Sweden. J Natl Cancer Inst.

[24] Friberg E, Mantzoros CS, Wolk A (2007). Diabetes and risk of endometrial cancer: a population-based prospective cohort study. Cancer Epidemiol Biomarkers Prev.

[25] Lucenteforte E, Bosetti C, Talamini R, Montella M, Zucchetto A, Pelucchi C, Franceschi S, Negri E, Levi F, La Vecchia C (2007). Diabetes and endometrial cancer: effect modification by body weight, physical activity and hypertension. Br J Cancer.

[26] Weiderpass E, Persson I, Adami HO, Magnusson C, Lindgren A, Baron JA (2000). Body size in different periods of life, diabetes mellitus, hypertension, and risk of postmenopausal endometrial cancer (Sweden). Cancer Causes Control.

[27] Soler M, Chatenoud L, Negri E, Parazzini F, Franceschi S, la Vecchia C (1999). Hypertension and hormone-related neoplasms in women. Hypertension.

[28] Evans AT 3rd, Gaffey TA, Malkasian GD Jr, Annegers JF (1980). Clinicopathologic review of 118 granulosa and 82 theca cell tumors. Obstet Gynecol.

[29] Koornstra JJ, Mourits MJ, Sijmons RH (2009). Management of extracolonic tumours in patients with Lynch syndrome. Lancet Oncol.

[30] Barrow E, Robinson L, Alduaij W (2009). Cumulative lifetime incidence of extracolonic cancers in Lynch syndrome: a report of 121 families with proven mutations. Clin Genet.

[31] Riegert-Johnson DL, Gleeson FC, Roberts M, Tholen K, Youngborg L, Bullock M, Boardman LA (2010). Cancer and Lhermitte-Duclos disease are common in Cowden syndrome patients. Hered Cancer Clin Pract.

[32] Pilarski R, Stephens JA, Noss R, Fisher JL, Prior TW (2011). Predicting PTEN mutations: an evaluation of Cowden syndrome and Bannayan-Riley-Ruvalcaba syndrome clinical features. J Med Genet.

[33] Win AK, Reece JC, Ryan S (2015). Family history and risk of endometrial cancer: a systematic review and meta-analysis. Obstet Gynecol.

[34] Bjørge T, Engeland A, Tretli S, Weiderpass E (2007). Body size in relation to cancer of the uterine corpus in 1 million Norwegian women. Int J Cancer.

[35] Felix AS, Weissfeld JL, Stone RA (2010). Factors associated with Type I and Type II endometrial cancer. Cancer Causes Control.

[36] Boruta DM 2nd, Gehrig PA, Fader AN (2009). Management of women with uterine papillary serous cancer: a Society of Gynecologic Oncology (SGO) review. Gynecol Oncol.

[37] Feldman S Evaluation of the endometrium for malignant or premalignant disease. Women who should undergo evaluation for endometrial hyperplasia or endometrial cancer. Literature review current through: Mar 2015.

[38] Lindor NM, Petersen GM, Hadley DW (2006). Recommendations for the Care of Individuals With an Inherited Predisposition to Lynch syndrome: A Systematic Review. JAMA.

[39] NCCN Clinical practice guidelines in oncology. National Comprehensive Cancer Network (NCCN).

[40] Lu KH, Schmeler KM Endometrial and ovarian cancer screening and prevention in women with Lynch syndrome (hereditary nonpolyposis colorectal cancer), UpToDate, topic last updated Feb17, 2014.

[41] (2006). American College of Obstetricians and gynecologists. ACOG Committee Opinion No. 336,: tamoxifen and uterine cancer. Obstet gynecol.

[42] Fisher B, Costantino JP, Wickerham DL (2005). Tamoxifen for the prevention of breast cancer: current status of the National Surgical Adjuvant Breast and Bowel Project P-1 study. J Natl Cancer Inst.

[43] Smith-Bindman R, Kerlikowske K, Feldstein VA, Subak L, Scheidler J, Segal M, Brand R, Gracy D (1998). Endovaginal ultra-sound to exclude endometrial cancer and other endometrial abnormalities. JAMA.

[44] Karlsson B, Granberg S, Wikland M, Ylostalo P, Torvid K, Marsal K, Valentin L (1995). Transvaginal ultrasonography of the endometrium in women with postmenopausal bleeding – a Nordic multicenter study. Am J Obstet Gynecol.

[45] Prendergast EN, Misch E, Chou YA, Roston A, Patel A (2014). Insufficient endometrial biopsy results in women with abnormal uterine bleeding. Obstet Gynecol.

[46] Goldstein SR, Nachtigall M, Snyder JR, Nachtigall L (1990). Endometrial assessment by vaginal ultrasonography before endometrial sampling in patients with postmenopausal bleeding. Am J Obstet Gynecol.

[47] (2009). American College of Obstetricians and Gynecologists. The role of transvaginal ultrasonography in the evaluation of postmenopausal bleeding. ACOG Committee Opinion No. 440. Obstet Gynecol.

[48] Goldstein RB, Bree RL, Benson CB, Benacerraf BR, Bloss JD, Carlos R, Fleischer AC, Goldstein SR, Hunt RB, Kurman RJ, Kurtz AB, Laing FC, Parsons AK, Smith-Bindman R, Walker J (2001). Evaluation of the woman with postmenopausal bleeding: Society of Radiologists in Ultrasound-Sponsored Consensus Conference statement. J Ultrasound Med.

[49] Tabor A, Watt HC, Wald NJ (2002). Endometrial thickness as a test for endometrial cancer in women with postmenopausal vaginal bleeding. Obstet Gynecol.

[50] Breijer MC, Peeters JA, Opmeer BC, Clark TJ, Verheijen RH, Mol BW, Timmermans A (2012). Capacity of endometrial thickness measurement to diagnose endometrial carcinoma in asymptomatic postmenopausal women: a systematic review and meta-analysis. Ultrasound Obstet Gynecol.

[51] Smith-Bindman R, Weiss E, Feldstein V (2004). How thick is too thick?. When endometrial thickness should prompt biopsy in postmenopausal women without vaginal bleeding. Ultrasound Obstet Gynecol.

[52] Dueholm M, Moller C, Rydbjerg S (2014). An ultrasound algorithm for identification of endometrial cancer. Ultrasound Obstet Gynecol.

[53] Munro MG, Critchley HO, Broder MS, Fraser IS (2011). IFGO Working Group on Menstrual Disorders. IFGO classification system (PALM-COEIN) for causes of abnormal uterine bleeding in nongravid women of reproductive age. Int J Gynaecol Obstet.

[54] Lipscomb GH, Lopatine SM, Stoval TG, Ling FW (1994). A randomised comparison of the Pipelle, Accurette, and Explora endometrial sampling devices. Am J Obstet Gynecol.

[55] Krampl E, Bourne T, Hurlen-Solbakken H, Istre O (2001). Transvaginal ultrasonography sonohysterography and operative hysteroscopy for the evaluation of abnormal uterine bleeding. Acta Obstet Gynecol Scand.

[56] de Kroon CD, de Bock GH, Dieben SW, Jansen FW (2003). Saline contrast hysterosonography in abnormal uterine bleeding: a systematic review and meta-analysis. BJOG.

[57] Torres ML, Weaver AL, Kumar S, Uccella S, Famuyide AO, Cliby WA, Dowdy SC, Gostout BS, Mariani A (2012). Risk factors for developing endometrial cancer after benign endometrial sampling. Obstet Gynecol.

[58] Dijkhuizen FP, Mol BW, Brölmann HA, Heintz AP (2000). The accuracy of endometrial sampling in the diagnosis of patients with endometrial carcinoma and hyperplasia: a meta-analysis. Cancer.

[59] Clark TJ, Mann CH, Shah N, Khan KS, Song F, Gupta JK (2002). Accuracy of outpatient endometrial biopsy in the diagnosis of endometrial cancer: a systematic quantitative review. BJOG.

[60] Guido RS, Kanbour-Shakir A, Rulin MC, Christopherson WA (1995). Pipelle endometrial sampling. Sensitivity in the detection of endometrial cancer. J Reprod Med.

[61] Clark TJ, Mann CH, Shah N, Khan KS, Song F, Gupta JK (2001). Accuracy of outpatient endometrial biopsy in the diagnosis of endometrial hyperplasia. Acta Obstet Gynecol Scand.

[62] Cooper JM, Erickson ML (2000). Endometrial sampling techniques in the diagnosis of abnormal uterine bleeding. Obstet Gynecol Clin North Am.

[63] Gimpelson RJ, Rappold HO (1988). A comparative study between panoramic hysteroscopy with directed biopsies and dilatation and curettage. A review of 276 cases. Am J Obstet Gynecol.

[64] Epstein E, Ramirez A, Skoog L, Valentin L (2001). Dilatation and curettage fails to detect most focal lesions in the uterine cavity in women with post menopausal bleeding. Acta Obstet Gynaecol Scand.

[65] Larson DM, Johnson KK, Broste SK, Krawisz BR, Kresl JJ (1995). Comparison of D&C and office endometrial biopsy in predicting final histopathologic grade in endometrial cancer. Obstet Gynecol.

[66] Deckardt R, Lueken RP, Gallinat A (2002). Comparison of transvaginal ultrasound, hysteroscopy, and dilatation and curettage in the diagnosis of abnormal vaginal bleeding and intrauterine pathology in perimenopausal and postmenopausal women. J Am Assoc Gynecol Laparosc.

[67] Trimble CL, Method M, Leitao M (2012). Management of endometrial precancers. Obstet Gynecol.

[68] Lee S, Andreotti R, Angtuaco T American Coolege of Radiology Appropriateness Criteria: endometrial cancer of the uterus. Reviewed 2007. http://www.acr.org/SecondaryMainMenuCategiries/quality_safety/app_criteria.aspx.

[69] Barakat R, Markman M, Randall M (2009). Principles and Practice of Gynecologic Oncology.

[70] Vladareanu R, Bohîlțea R (2006). Cancerul de corp uterin. In: Vladareanu R. Obstetrică și Ginecologie Clinică.

[71] Hsieh CH, ChangChien CC, Lin H (2002). Can a preoperative CA 125 level be a criterion for full pelvic lymphadenectomy in surgical staging of endometrial cancer?. Gynecol Oncol.

[72] Dotters DJ (2000). Preoperative CA 125 in endometrial cancer: is it useful?. Am J Obstet Gynecol.

[73] Todo Y, Sakuragi N, Nishida R (2003). Combined use of magnetic resonance imaging, CA 125 assay, histologic type, and histologic grade in the prediction of lymph node metastasis in endometrial carcinoma. Am J Obstet Gynecol.

[74] Powell JL, Hill KA, Shiro BC (2005). Preoperative serum CA-125 levels in treating endometrial cancer. J Reprod Med.

